# Correlation and variation of cuff inflating volumes and pressures in different adult models of laryngeal mask: a prospective randomized trial

**DOI:** 10.1186/s12871-020-01028-4

**Published:** 2020-05-07

**Authors:** Narut Ruananukun, Jittiya Watcharotayangul, Suchaya Jeeranukosol, Rojnarin Komonhirun

**Affiliations:** Department of Anesthesiology, Faculty of Medicine Ramathibodi Hospital, Mahidol University, 270 Rama VI Road, Rajchathewi, Bangkok, 10400 Thailand

**Keywords:** Laryngeal mask, Cuff inflating volume, Intracuff pressure

## Abstract

**Background:**

Hyperinflation of laryngeal mask cuffs may carry the risk of airway complications. The manufacturer recommends inflating cuff until the intracuff pressure reaches 60 cmH_2_O, or inflate with the volume of air to not exceed the maximum recommended volume. We prospectively assessed the correlation of cuff inflating volumes and pressures, and the appropriated the cuff inflating volumes to generate an intracuff pressure of 60 cmH_2_O in the adult laryngeal masks from different manufacturers.

**Methods:**

Two groups of 80 patients requiring laryngeal mask size 3 and 4 during general anesthesia were randomized into 4 subgroups for each size of the laryngeal mask: Soft Seal® (Portex®), AuraOnce™ (Ambu®), LMA-Classic™ (Teleflex®) and LMA-ProSeal™ (Teleflex®). After insertion, the cuff was inflated with 5-ml increments of air up to the maximum recommended volume. After each 5-ml intracuff pressure was measured, the volume of air that generated the intracuff pressure of 60 cmH_2_O was recorded**.**

**Results:**

Mean (SD) volume of air required to achieve the intracuff pressure of 60 cmH_2_O in Soft Seal®, AuraOnce™, LMA-Classic™, LMA-ProSeal™ laryngeal mask size 3 were 11.80(1.88), 9.20(1.88), 8.95(1.50) and 13.50(2.48) ml, respectively, and these volumes in laryngeal mask size 4 were 14.45(4.12), 12.55(1.85), 11.30(1.95) and 18.20(3.47) ml, respectively. The maximum recommended volume resulted in high intracuff pressures (> 60 cmH_2_O) in all laryngeal mask types and sizes studied.

**Conclusion:**

Pressure-volume curves of adult laryngeal masks are all in sigmoidal shape. Cuff designs and materials can effect pressure and volume correlation. Approximately half of the maximum recommended volume is required to achieve the intracuff pressure of 60 cmH_2_O except LMA-ProSeal™ which required two-thirds of the maximum recommended volume.

**Trial registration:**

Thai Clinical Trials Registry, TCTR20150602001, May 28, 2015.

## Background

The laryngeal mask hyperinflation related complications range from sore throat to more serious complications such as paralysis of the vocal cord, arytenoid cartilages dislocation, recurrent laryngeal nerve injury and hypoglossal nerve injury [1–6]. The manufacturers recommend inflating the laryngeal mask cuff until the intracuff pressure reaches 60 cmH_2_O or to inflate with the volume of air not exceeding the maximum recommended volume (size 3, 20 ml; size 4, 30 ml) if a manometer is not available [7–11]. It is common practice to inflate the laryngeal mask cuff without using a manometer.

The studies of pressure–volume relationship in pediatric laryngeal masks show that approximately half the recommended maximum volume produced a laryngeal mask cuff pressure above the recommended pressure of 60 cmH_2_O [12–14]. The previous study of size 4 LMA-ProSeal™ showed that inflation with a filling volume of 15–20 ml was the proper position and was optimal for ventilation without leakage [15]. Furthermore, inflation of size 4 LMA-ProSeal™ with the maximum recommended volume (30 ml) does not improve the seal pressure and may actually increase the risk of gastric insufflation [15]. However, they are only one adult model and pediatric laryngeal masks studies. There are few studies of cuff inflating volumes and pressures in different adult models of laryngeal mask.

The aims of this study were to assess the correlation of cuff inflating volumes and pressures, and to evaluate the cuff inflating volumes that generate an intracuff pressure of 60 cmH_2_O in adult laryngeal mask (size 3 and 4) from different manufacturers which are made with varying cuff designs and materials.

## Methods

**Fig. 1:**
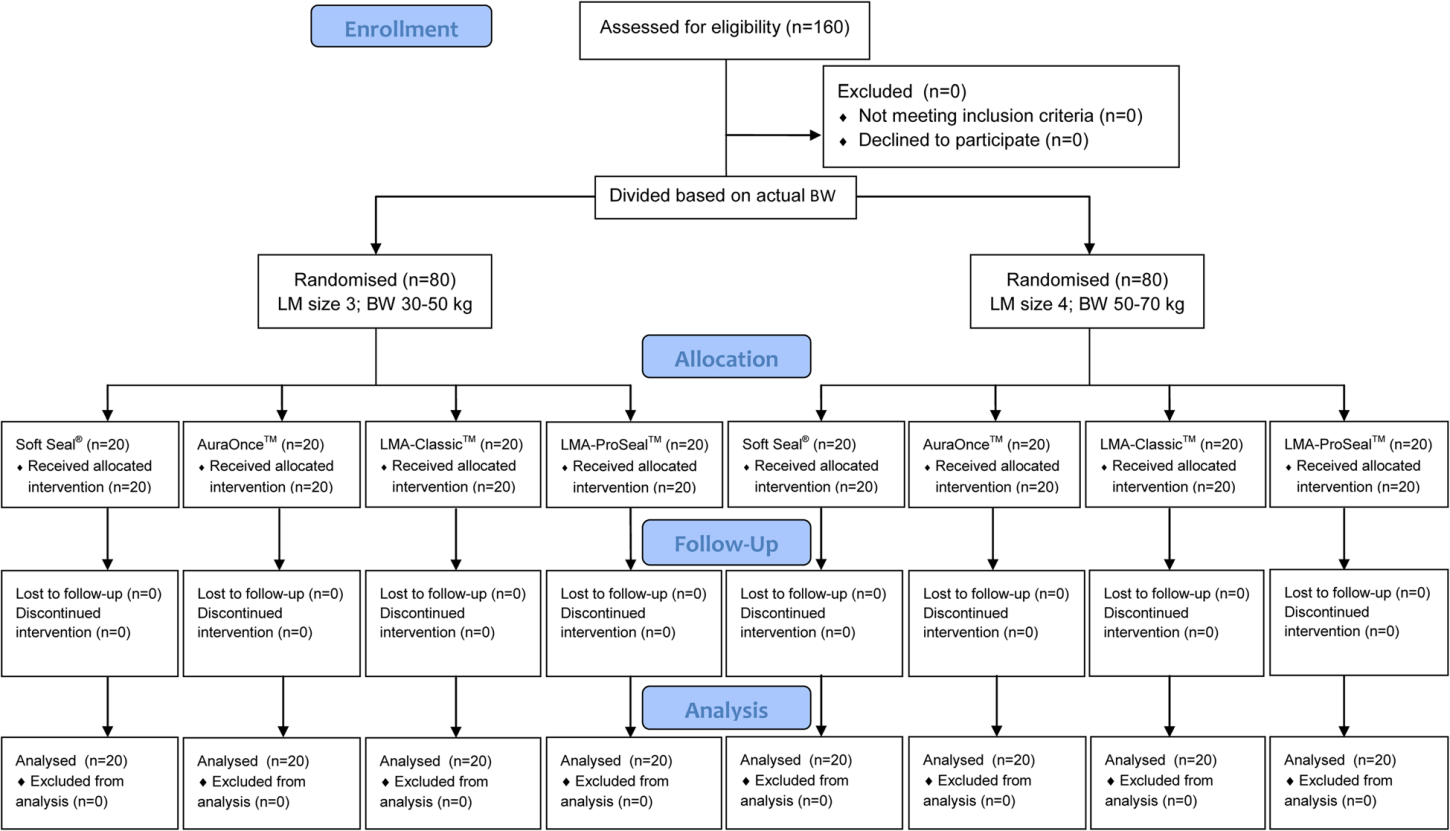
CONSORT Flow Diagram; LM = laryngeal mask

This was a prospective randomized trial. The study was approved by the Ethics Committee of Faculty of Medicine Ramathibodi Hospital, Mahidol University (10–57-33). Written informed consent was obtained from each patient on the day before the operation. This study adhered to the applicable CONSORT guidelines. We studied 160 Thai patients who were scheduled for all types of surgery under general anesthesia with the laryngeal mask. The patients were older than 18 years of age, weighed 30–70 kg, ASA physical status I-III, and were NPO for 6 h before the operation. Patients with any pathology of the neck, upper respiratory or upper alimentary tract, risk of aspiration, body mass index (BMI) > 30 kg. m^− 2^ and predictors of difficult airway such as restricted mouth opening (< 3 cm of interincisor distance) were excluded. The patients were divided into 2 groups by actual body weight (30–50 kg for laryngeal mask size 3, 50–70 kg for laryngeal mask size 4) and then the patients in each group were randomized by computer into 4 subgroups of laryngeal mask type [Soft Seal® laryngeal mask (Portex® Smiths Medical International Ltd., Hythe Kent, UK); AuraOnce™ laryngeal mask (Ambu® Inc. Glen Burnie, MD, USA); LMA-Classic™ (Teleflex® The Laryngeal Mask Company Ltd., Victoria, Seychelles); LMA-ProSeal™ (Teleflex® The Laryngeal Mask Company Ltd., Victoria, Seychelles)] as Fig. 1 and sealed in opaque envelopes.

**Fig. 2:**
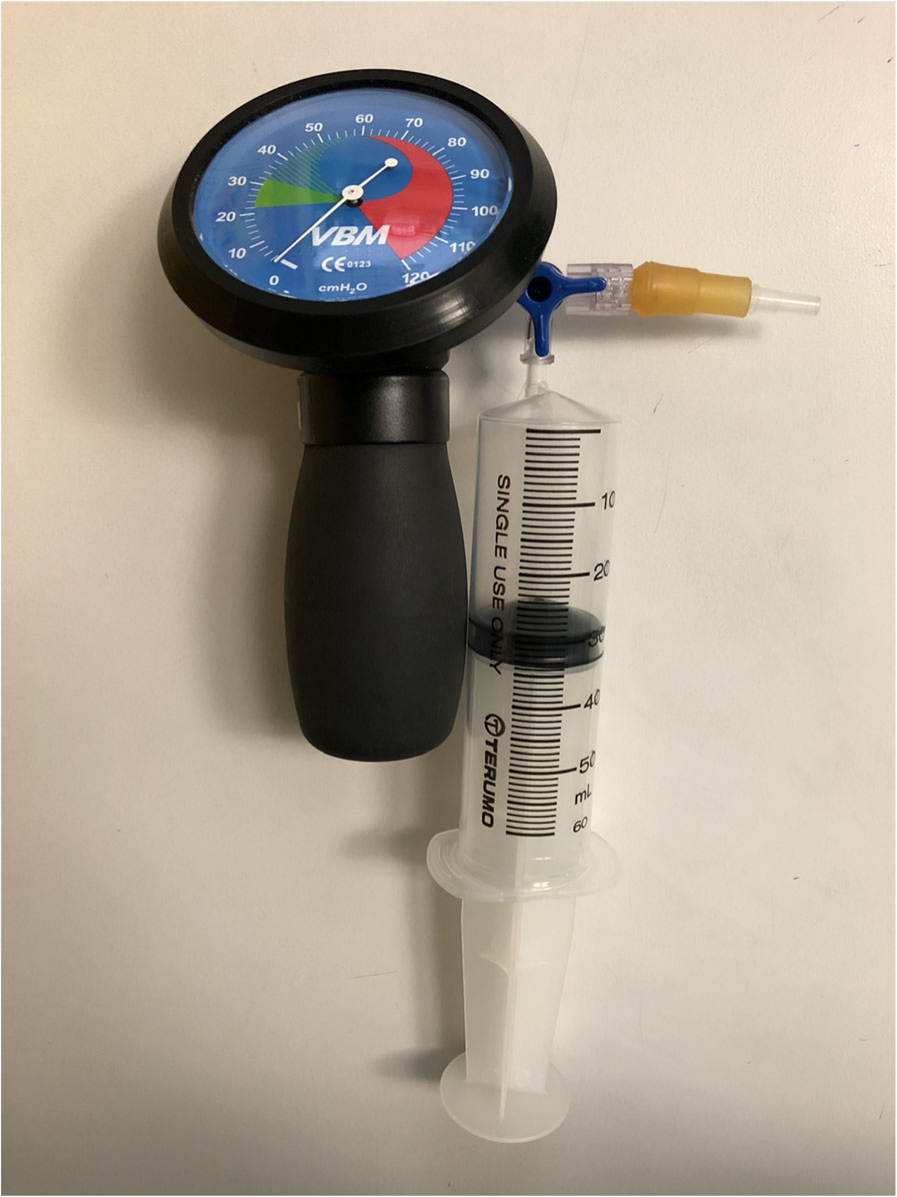
Closed system manometer

After standard monitoring such as electrocardiography, noninvasive blood pressure device and pulse oximeter were applied, general anesthesia was conducted with propofol 2.5 mg.kg^− 1^ and fentanyl 1 mcg.kg^− 1^ intravenously then the randomized laryngeal mask was inserted after appropriate conditions for laryngeal mask insertion obtained. The position of the inflated laryngeal mask was checked by assuring there was no air leakage and there was good chest movement when positive pressure ventilation was performed at an airway pressure of around 20 cmH_2_O. After the laryngeal mask was fixed with adhesive tape and the vital signs were stable, the ventilation was stopped. The laryngeal mask cuff was fully deflated and connected with a closed system manometer which was composed of a three-way stopcock, 50 ml syringe and manometer (Cuff Inflator Pressure Gauge; VBM, Medizintechnik GmbH, Germany), as shown in Fig. 2. The laryngeal mask cuff was inflated with 5-ml increments of air up to the maximum recommended volume or until the intracuff pressure of 120 cmH_2_O. After each 5-ml of intracuff pressure was measured and the inflating volume that generated intracuff pressure of 60 cmH_2_O was recorded, in the end, all intracuff pressures were adjusted to 60 cmH_2_O.

### Statistical analysis

We performed a pilot study on 10 patients each with laryngeal mask size 3 and 4 (total of 20 patients) to find the mean of cuff inflation volume required to achieve the appropriate intracuff pressure of 60 cmH_2_O. The standard deviation (SD) of cuff inflation volume in laryngeal mask size 3 and 4 was 2.2 ml and 2.3 ml, respectively. We defined the allowable error of volume to be 1 ml, with a 95% confidence interval and we worked under the assumption that all laryngeal mask has similar accuracy. The pilot study revealed that sample sizes for laryngeal mask size 3 and 4 are 19 and 20 samples respectively. Therefore sample sizes of each type of laryngeal mask included 39 samples, and the total samples sizes of the 4 types of laryngeal mask consisted of 156 samples. We included 160 patients to compensate for any possible dropouts.

Data were analyzed using SPSS software package version 18.0 (SPSS, Chicago, IL, USA). Mean and standard deviation (SD) were calculated for continuous variables such as age, weight, and height. Frequency and percentages were calculated for categorical variables such as sex and ASA physical status. Median, maximum-minimum values or interquartile range were calculated for discrete variables**.** The data were reported by different types and sizes of laryngeal mask. The relationship between cuff inflation volume and intracuff pressure in the laryngeal mask were fitted by linear regression line. Qualitative data were analyzed using Chi-square test, Fisher exact test and Monte Carlo test. Quantitative data were analyzed using Kruskal-Wallis H test. A *p*-value of less than 0.05 was indicated to be statistically significant.

## Results

There were no failed insertion, dislodgement of laryngeal mask, airway obstruction or pulmonary aspiration during anesthesia; therefore, all patients enrolled in the study were included in the analysis. Patient characteristics and laryngeal mask size and type are shown in Table 1.

**Table 1:** Characteristics of patients receiving the four types of laryngeal mask

Laryngeal mask		Soft Seal® (*n* = 20)	AuraOnce™ (*n* = 20)	LMA-Classic™ (*n* = 20)	LMA-ProSeal™ (*n* = 20)	*p* value
Size 3	Age (years)	44.2 (16.08)	41.15 (15.90)	47.25 (20.30)	52.65 (17.31)	0.203
	Male/Female, n	1/19	1/19	0/20	0/20	> 0.999
	Body weight (kg)	47.4 (3.39)	47.6 (2.85)	47.65 (3.27)	49.55 (4.37)	0.703
	Height (cm)	155.3 (3.16)	155.8 (5.38)	154.4 (4.89)	154.4 (5.43)	0.741
	BMI (kg.m^− 2^)	19.67 (1.58)	19.65 (1.54)	20.03 (1.7)	20.82 (1.96)	0.112
	ASA physical status, n (%)					0.188
	1	11 (55%)	11 (55%)	7 (35%)	8 (40%)	
	2	8 (40%)	8 (40%)	10 (50%)	5 (25%)	
	3	1 (5%)	1 (5%)	3 (15%)	7 (35%)	
Size 4	Age (years)	50.10 (11.85)	52.4 (14.16)	55.75 (13.85)	52.1 (15.98)	0.645
	Male/Female, n	1/19	1/19	2/18	6/14	0.078
	Body weight (kg)	62.7 (6.17)	59.2 (5.85)	62.6 (7.17)	59.05 (5.51)	0.093
	Height (cm)	157.4 (5.83)	155.5 (5.72)	159.75 (5.37)	160.45 (5.84)	0.029*
	BMI (kg.m^−2^)	25.34 (2.59)	24.51 (2.4)	24.54 (2.72)	23.03 (2.84)	0.054
	ASA physical status, n (%)					0.409
	1	8 (40%)	4 (20%)	9 (45%)	5 (25%)	
	2	9 (45%)	13 (65%)	8 (40%)	10 (50%)	
	3	3 (15%)	3 (15%)	3 (15%)	5 (25%)	

Data are presented as mean (SD) unless stated otherwise

The volume of air generated the intracuff pressure in Soft Seal®, AuraOnce™, LMA-Classic™, LMA-ProSeal™ laryngeal mask size 3 and 4 are shown in Table 2.

**Table 2:** Cuff volume and pressure at different values

Laryngeal mask			Soft Seal® (*n* = 20)	AuraOnce™ (*n* = 20)	LMA-Classic™ (*n* = 20)	LMA-ProSeal™ (*n* = 20)
Size 3	Cuff pressure at (cmH_2_O)	5 ml	24.90 (4.78)	27.85 (10.54)	33.10 (10.81)	17.75 (7.42)
		10 ml	50.40 (9.94)	72.20 (22.40)	72.80 (13.05)	43.15 (10.21)
		15 ml	89.55 (16.81)	109.0 (14.58)	113.60 (12.36)	76.65 (18.51)
		20 ml	115.85 (90.90)	120.0 (0.00)	120.0 (0.00)	107.40 (15.81)
	Cuff volume at (ml)	60 cmH_2_O	11.80 (1.88)	9.20 (1.88)	8.95 (1.50)	13.50 (2.48)
Size 4	Cuff pressure at (cmH_2_O)	5 ml	22.55 (9.13)	23.20 (7.10)	24.15 (5.79)	14.55 (4.30)
		10 ml	43.80 (13.63)	46.20 (10.97)	53.95 (9.64)	29.70 (8.78)
		15 ml	68.15 (20.89)	84.65 (17.35)	88.15 (18.49)	49.10 (11.77)
		20 ml	96.60 (25.01)	116.21 (10.20)	113.0 (13.04)	76.45 (20.42)
		25 ml	106.71 (19.73)	120.0 (0.00)	120.0 (0.00)	104.25 (16.99)
		30 ml	–	–	–	114.82 (5.23)
	Cuff pressure at (ml)	60 cmH_2_O	14.45 (4.12)	12.55 (1.85)	11.30 (1.95)	18.20 (3.47)

Data are presented as mean (SD)

**Fig. 3:**
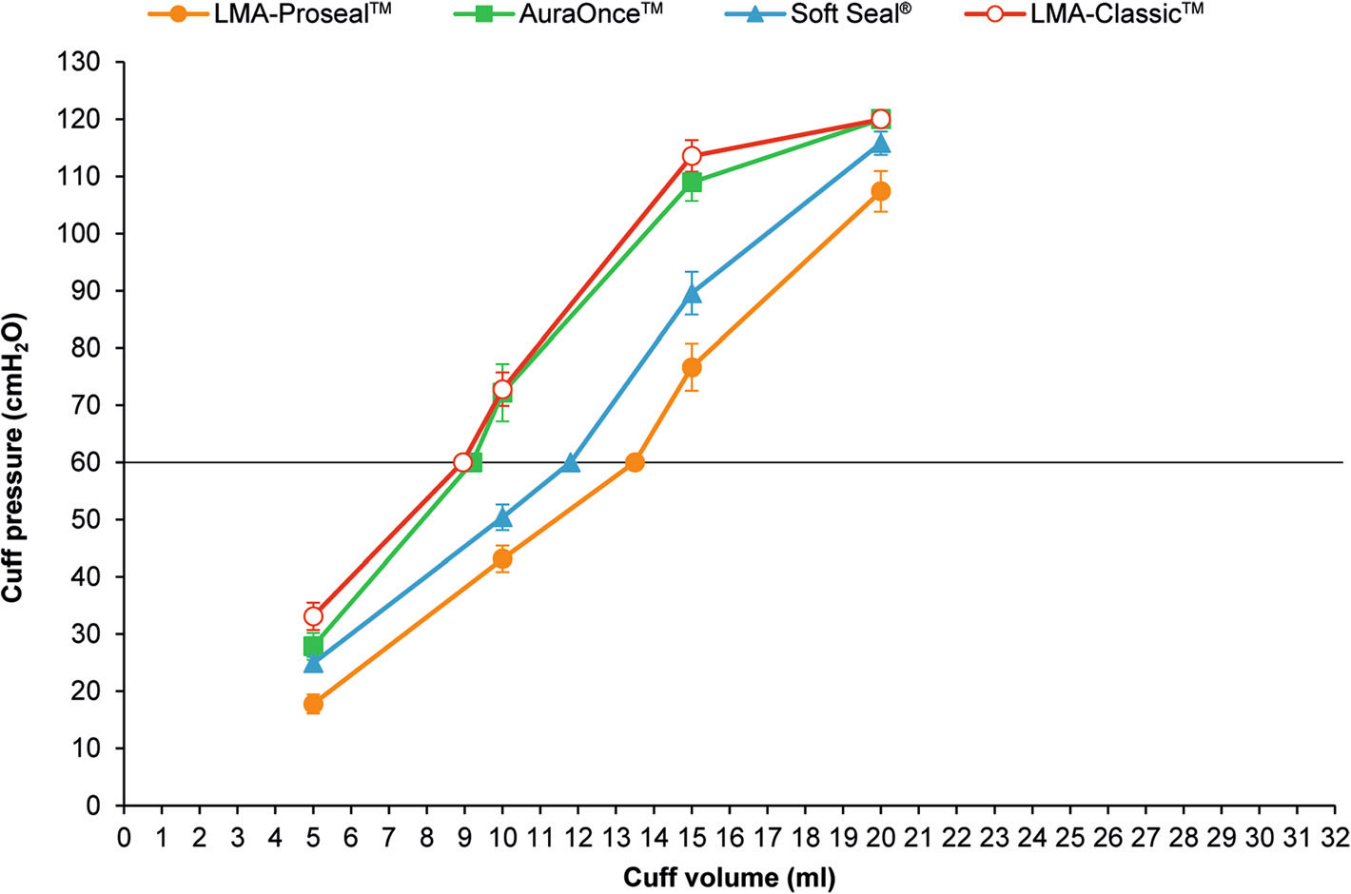
Pressure-volume curves for the four types of laryngeal mask size 3: Soft Seal® (), AuraOnce™ (), LMA-Classic™ (), LMA-ProSeal™ ()

**Fig. 4:**
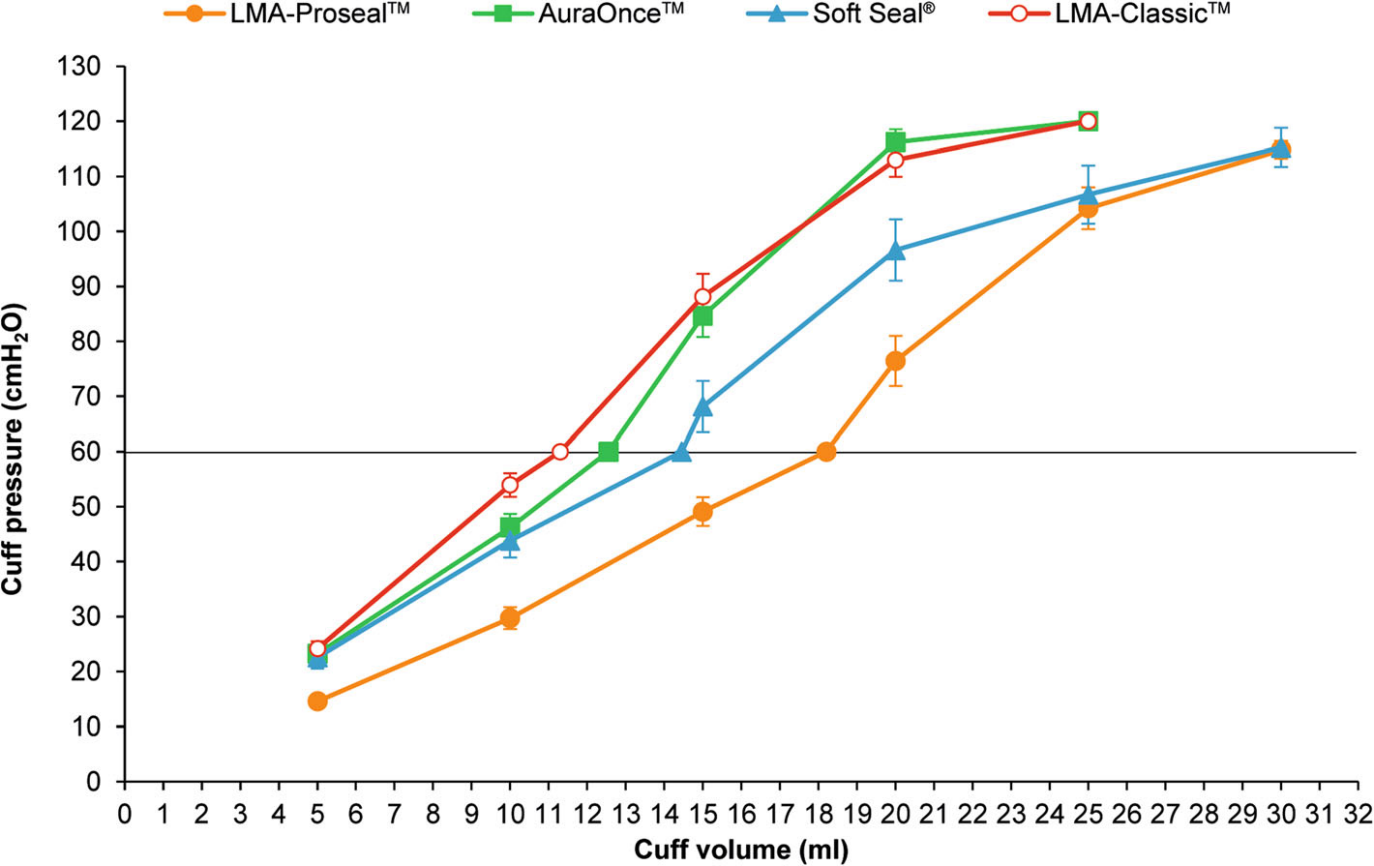
Pressure-volume curves for the four types of laryngeal mask size 4: Soft Seal® (), AuraOnce™ (), LMA-Classic™ (), LMA-ProSeal™ ()

The laryngeal mask intracuff pressure-volume relationships are depicted in Table 2, Fig. 3 and Fig. 4. The pressure-volume graphs for each laryngeal mask size and type (Figs. 3, 4) demonstrate the maximum pressure limits (60 cmH_2_O) being exceeded well below the recommended maximum inflation volume; approximately half of the maximum inflation volume for each laryngeal mask resulted in pressures around 60 cmH_2_O, except LMA-ProSeal™ which required two-thirds of the maximum recommended volume.

## Discussion

Pressure-volume curves of the four types of adult laryngeal mask are similar sigmoidal (S-shape) curves but they have different levels of pressure and volume correlations which may be caused by their cuff designs and materials. These result agree with the previous studies of pediatric laryngeal masks [12, 13].

This study assessed pressure and volume correlation of adult laryngeal masks in the volume range as recommended by the manufacturer. Inflating the laryngeal mask from a fully deflated state to only approximately half to two-thirds of the recommended maximum volume caused the laryngeal mask cuff pressure to rise above the recommended pressure of 60 cmH_2_O. This is in line with previous studies showing hyperinflation of the laryngeal mask cuff when applying the maximum recommended volume [10–15].

This study shows LMA-ProSeal™ requires approximately two-thirds of the recommended maximum volume to achieve an intracuff pressure of 60 cmH_2_O, but the other types require approximately half of the recommended maximum volume to achieve this pressure. So the type of laryngeal mask affects pressure and volume correlation due to cuff sizes, designs and materials.

In clinical practice, the intracuff pressure is not routine monitoring. Our results show that small volume of air which was inflated laryngeal mask cuff, it can easily generate high intracuff pressure.

Although the manufacturers suggest that the inflation volumes should not be above the maximum recommended volumes and an intracuff pressure of 60 cm H_2_O can be achieved by the lower volumes [6–10], a lot of practitioners miss this concept. So, the manufacturer’s recommendations should be emphasized that the manometer should be used to monitor the intracuff pressure to prevent excessive intracuff pressure and its complications.

In this study, we found some limitations. First, there were not an equal number of males and females. Female patients were more common. The results may be different in other groups studied.

Second, we used clinical signs for confirmation of laryngeal mask position based on there being no leakage during ventilation at an airway pressure of 20 cmH_2_O and have good chest movement. It may be better if the fibreoptic assessment were used to confirm the position [15]. Nevertheless, our method is more common and it can be applied to the clinical practice.

Third, we did not study the minimum cuff inflation volume of laryngeal mask that achieves adequate ventilation. Further studies about this volume and intracuff pressure may be useful for cuff inflation technique.

Finally, this study measured the intracuff pressure but this pressure may not be transmitted pressure to adjacent mucosa in the pharynx and larynx. Further studies using microchip sensors attached to laryngeal mask surfaces for measuring transmitted pharyngeal mucosal pressure may be beneficial in different models and sizes of laryngeal masks because cuff design and material may affect the transmitted pressure in different locations [16]**.**

## Conclusion

Pressure-volume curves of adult laryngeal masks are all in sigmoidal shape. Cuff designs and materials can effect pressure and volume correlation. Approximately half of the maximum recommended volume is required to achieve an intracuff pressure of 60 cmH_2_O except Proseal LMA which required two-thirds of the maximum recommended volume. However, the manometer should be used to guide inflation in adult LMA if the inflation volume is higher than that mentioned above or use LMA with cuff pressure pilot valve which enables user to monitor the intracuff pressure.

## Data Availability

The datasets used and analyzed during the current study are available from the corresponding author upon reasonable request.

## Abbreviations

ASA: American Society of Anesthesiologists
NPO: Nil per os
LMA: Laryngeal mask airway
